# Factors associated with patient visits to the emergency department for asthma therapy

**DOI:** 10.1186/1471-2466-12-80

**Published:** 2012-12-17

**Authors:** Hamdan AL-Jahdali, Ahmed Anwar, Abdullah AL-Harbi, Salim Baharoon, Rabih Halwani, Abdulllah Al Shimemeri, Saleh Al-Muhsen

**Affiliations:** 1 Department of Medicine, Pulmonary Division-ICU, King Saud University for Health Sciences, Riyadh, Saudi Arabia; 2 Department of Epidemiology and Biostatistics, College of Public Health and Health, Informatics, King Saud bin Abdulaziz University for Health Sciences, Riyadh, Saudi Arabia; 3 Asthma Research Chair and Prince Naif Center for Immunology Research, Department of Paediatrics, College of Medicine, King Saud University, Riyadh, Saudi Arabia; 4 Head of Pulmonary Division, Medical Director of Sleep Disorders Center, Adjunct professor McGill University, King Saud University for Health Sciences, King Abdulaziz Medical City, Riyadh, Riyadh, Saudi Arabia

**Keywords:** Asthma, Control, Inhaled cortisone, Emergency department

## Abstract

**Background:**

Acute asthma attacks remain a frequent cause of emergency department (ED) visits and hospital admission. Many factors encourage patients to seek asthma treatment at the emergency department. These factors may be related to the patient himself or to a health system that hinders asthma control. The aim of this study was to identify the main factors that lead to the frequent admission of asthmatic patients to the ED.

**Methods:**

A cross-sectional survey of all the patients who visited the emergency room with bronchial asthma attacks over a 9-month period was undertaken at two major academic hospitals. The following data were collected: demographic data, asthma control in the preceding month, where and by whom the patients were treated, whether the patient received education about asthma or its medication and the patients’ reasons for visiting the ED.

**Result:**

Four hundred fifty (N = 450) patients were recruited, 39.1% of whom were males with a mean age of 42.3 ± 16.7. The mean duration of asthma was 155.90 ± 127.13 weeks. Approximately half of the patients did not receive any information about bronchial asthma as a disease, and 40.7% did not receive any education regarding how to use asthma medication. Asthma was not controlled or partially controlled in the majority (97.7%) of the patients preceding the admission to ED. The majority of the patients visited the ED to receive a bronchodilator by nebuliser (86.7%) and to obtain oxygen (75.1%). Moreover, 20.9% of the patients believed that the ED managed them faster than the clinic, and 21.1% claimed that their symptoms were severe enough that they could not wait for a clinic visit. No education about asthma and uncontrolled asthma are the major factors leading to frequent ED visits (three or more visits/year), p-value = 0.0145 and p-value = 0.0003, respectively. Asthma control also exhibited a significant relationship with inhaled corticosteroid ICS use (p-value =0.0401) and education about asthma (p-value =0.0117).

**Conclusion:**

This study demonstrates that many avoidable risk factors lead to uncontrolled asthma and frequent ED visits.

## Background

Asthma is a common condition that affects 5-10% of the population. The incidence and prevalence of asthma have increased during the past 20 years [1,2]. The prevalence of bronchial asthma among Saudi patients is approximately 20-25% [2,3]. Poor asthma control remains a frequent cause of emergency department (ED) presentation and hospital admission [4]. The cost of uncontrolled asthma care is substantial. For example, the utilisation of the emergency department for asthma management accounts for almost one-third of all asthma costs in the United States [5].

There are many factors that lead patients to visit the ED. The most common reported factors include asthma severity, poor compliance, the inappropriate use of inhalers, incorrect perceptions about bronchial asthma as a disease or about its medication, the cost of medication, lack of an asthma action plan, comorbidities, over reliance on short acting bronchodilators, pollution and changes in the weather, the patient’s level of education and low socioeconomic status [5-19].

Reducing the use of the ED for acute asthma treatment remains a major goal of asthma management that is recommended by all guidelines [20-23]. It is not clear why many patients in our community still visit the ED and depends on the ED as their primary if not sole source of care. It is important to understand the factors associated with asthma-related ED visits in order to reduce the use of ED resource utilization for asthma treatment. There are many factors that encourage patients to seek asthma treatment at the ED and these factors may be different from one society to another. It is very important to identify characteristics of the patients and deficiencies in our health care delivery system related factors causing poor asthma control and frequent visits to the emergency department (ED). The objective of this study is to evaluate the most important factors associated with the increased usage of the emergency department in our population.

## Methods

This was a cross-sectional study conducted at the King Abdulaziz Medical City- King Fahad National Guard Hospital in Riyadh (KAMC-KFNGH) and the King Khalid University Hospital (KKUH). We enrolled patients with diagnosis of asthma who visited the ED for asthma management between August 2010 and March 2011. The enrolled patient must have a documented diagnosis of bronchial asthma as diagnosed by their primary treating physician and on prescribed inhaled corticosteroid (ICS) for at least the last three months. We excluded patients with undocumented diagnosis of bronchial asthma and not on ICS as per their medical record. This study was approved by the IRBs of both hospitals (Ref. IRBC/123/11). During ED visit, the trained co-investigator collected information about demographic data, the duration of the illness, the medication used for asthma therapy and if the patient received any formal asthma education about asthma as a disease, how to use their inhaler devices and by whom. The patients were asked about regular visits to outpatient clinics, where they followed up, and how many times they visited the emergency department or were hospitalised over the last year. Co-investigators also verify this information by reviewing the medical record of each patient and assess asthma control over the last month by administering validated published Arabic version of Asthma Control Test (ACT) [24].

### Statistical analysis

The collected data were transferred and analyzed using SAS® version 9.2 (SAS Institute Inc., Cary, NC). Descriptive statistics, such as means, standard deviations, or median were used to summarize age and duration of asthma disease. Percentages were also used to summarize gender, ICS use, follow up with clinics, education level, educated about medication, educated about asthma, and reasons for visiting the ED. Mann–Whitney test was used to compare the distributions of asthma disease duration across number of asthma-related ED visits (< 3 vs. ≥ 3). Chi squared tests were used to test the associations between gender, ICS use, follow up with clinics, education level, educated about medication, and educated about asthma across asthma-related ED visits. Similar analysis used for asthma control test (ACT). Multiple logistic models were used to identify the risk factors that associated with three or more asthma-related ED visits. P-values less than 0.05 were considered significant. The odds ratios (ORs) with 95% CIs were reported to describe the strength of these associations.

## Results

Four hundred fifty (n = 450) asthma patients were enrolled in the study. Of the 450 asthma patients, 176 (39.1%) were males and 274 (60.9%) were females. The patient’s demographic and clinical characteristics are shown in Table 1. The mean patients’ age was 42.3 ±16.7 years, and the mean duration of asthma illness was 155.90 ± 127.13 weeks. Two hundred and seventy (60.0%) patients were regularly followed up with a physician, while 180 (40.0%) patients did not have any follow up arrangement after their initial diagnosis of asthma. Approximately half of the patients did not have any formal education about asthma 232 (51.6%), while 183 (40.7%) did not receive education about how to use the medication or the devices. Of 218 patients received information about asthma as a disease, 44.5% received this information from physicians, 7.8% received the information from asthma educators, and 4.7% received the information from a pharmacist. One hundred sixty five of the 450 patients (36.7%) visited the ED three or more per year. The patients’ asthma control for the last month before the ED visit was as follows: 23.4% of the patients with uncontrolled asthma (ACT score ≤ 15), 74.4% of the patients with partial controlled asthma (16 ≤ ACT score ≤ 23), 1.8% of the patients with complete controlled asthma (ACT score ≥ 24), and 0.5% of the patients with missing ACT score. When the patients were asked about the reason for the ED visit, the majority of the patients 86.7% indicated that receiving a nebulised bronchodilator was the major reason. Three hundred thirty-eight (75.1%) patients mentioned obtaining oxygen as their reason, while 20.9% believed that the ED treated their asthma faster, and 21.1% claimed that their asthma was severe enough that they could not wait to visit the clinic (Table 2). The majority of the patients, 74.7%, did not know what triggered their asthma, and 81.6% stopped all asthma therapy once they felt better.

**Table 1:** Patient demographics and clinical asthma characteristics (N = 450)

***Variable***	***Levels***	
Age, *(Mean ± SD)*		42.3 ± 16.7
Duration of illness in weeks (Mean ± SD)		155.90 ± 127.13)
Gender	*% Female*	60.9
Education level	*No school*	44.0
	*High school or less*	42.0
	*University*	13.8
	*Missing*	0.2
Employment Status	*Employee*	31.8
	*Student*	6.9
	*Housewife*	52.0
	*Non-employee*	4.7
	*Other*	4.4
	*Missing*	0.2
Follow up consistently with doctor		60.0
Follow-up clinic	*PHC/Family Medicine*	46.2
	*Pulmonary*	10.2
	*Internal Medicine*	1.8
	*Other*	1.8
	*No follow-up*	40.0
No education about asthma		51.6
No education about medication (devices)		40.7
ED visits	*<3*	61.3
	*≥3*	36.7
	*Missing*	2.0
Asthma control	*Uncontrolled*	23.3
	*Partially controlled*	74.4
	*Completely control*	1.8
	*Missing*	0.5

^*╦*^*All percentage rounded to one decimal.*

**Table 2:** Knowledge about asthma management and Reasons for visiting the ED (N = 450)

***Variable***	***%***
***Reason for ED visit***	
Visit ED primarily to obtain a bronchodilator	86.7
Visit ED to obtain oxygen	75.1
The severity of asthma doesn’t allow the patient to wait for a clinic visit.	21.1
Belief that the patient is treated faster in the ED	20.9
The ED is available 24 hours a day	19.1
The patient treated directly without delay	20.9
Medication given as nebulizer at ED is more useful	19.6
***Knowledge about asthma management***	
Take bronchodilator to relieve symptoms only	87.3
Stop ICS therapy when feel better	81.6
Believe long term use of inhaler unsafe	42.7
Believe continues use of inhaler cause dependence	35.1
Believe asthma therapy use its effect overtime	40.3
Does not know what trigger asthma symptoms	74.7
Does not know what should do during asthma attack	28.9

^*╦*^*All percentage rounded to one decimal.*

The asthma-related ED visits were classified on the basis of whether the asthma patient had three or more asthma-related ED visits. Table 3, shows the relationships between three or more asthma-related ED visits and the patient’s education level, education about asthma, ISC, and asthma control. Those who were not educated about asthma were more likely to visit the ED because of asthma than those who had been educated about asthma (42.7% versus 31.5%, p-value =0.0145). More of the patients with uncontrolled asthma (ACT score ≤ 15) than partially/fully controlled asthma (ACT score > 15) made three or more ED visits (52.4% versus 32.9%, p-value =0.0003). Table 4, shows the relationships between asthma control and patient’s demographic and clinical characteristics. There was a relationship between patient believe of needing oxygen for asthma therapy and three or more ED visits (40.5% versus 28.2%, p-value =0.0209), there was no relationship between visit ED primarily to obtain a bronchodilator and three or more ED visits (36.5% versus 43.3%, p-value =0.3081). Mann–Whitney test revealed there was no relationship between the duration of the disease and the number of ED visit (p = 0.3944). An education level higher than high school (p-value = 0.0071), an uncontrolled asthma (p-value = 0.0063), and irregular follow up with clinics (p-value = 0.0328) were highly associated with three or more asthma-related ED visits, after being controlled for gender, ICS use, education level, education about medication, and education about asthma (Table 5). As found in this study, the patients with university education were twice more likely to visit the ED than the patients with high school or not educated (OR: 2.359; 95% CI: 1.263, 4.407). The patients with uncontrolled asthma were twice as likely to come to the ED compared with the patients with controlled asthma (OR: 1.924; 95% CI: 1.203, 3.077). This study also showed that asthma control as determined by ACT had a significant relationship with ICS use (p-value = 0.0401), asthma education (p-value = 0.0117), ED visit primarily to obtain a bronchodilator (p-value = 0.0001), and ED visit to obtain oxygen (p-value = 0.0203). The distribution of uncontrolled asthma varied depending on patient ICS use (27.6% irregular, while 19.4% regular use). Those who had not been educated about asthma were more likely to have uncontrolled asthma than those who had been educated about asthma (28.1% versus 18.1%).

**Table 3:** The association between asthma-related ED visits and demographic and clinical characteristics (N = 441)

***Variable***	***Levels***	***<*3 visits**	**≥ 3 visits**	***p-value***
		**(n = 276)**	**(n = 165)**	
Gender	*% Male*	63.8	36.2	0.6721
	*Female*	61.8	38.2	
Regular ICS use	*Yes*	65.6	34.4	0.1880
	*No*	59.6	40.4	
Follow up with clinics	*Yes*	61.4	38.6	0.4688
	*No*	64.8	35.2	
Education level	*High school or less*	64.8	35.2	0.0133*
	*University*	48.4	51.6	
Educated about medication	*Yes*	64.5	35.5	0.3498
	*No*	60.1	39.9	
Educated about asthma	*Yes*	68.5	31.5	0.0145*
	*No*	57.3	42.7	
ACT	*Not controlled*	47.6	52.4	0.0003*
	*Partially/Full controlled*	67.1	32.9	

*The Chi-square statistic is significant at the .05 level.^*╦*^*All percentage rounded to one decimal.*

**Table 4:** The association between the asthma control test (ACT) and demographic and clinical characteristics (N = 448)

***Variable***	***Levels***	**Partially/Full controlled (n = 343)**	**Not controlled (n = 105)**	***p-value***
Gender	*% Male*	76.0	24.0	0.8220
	*Female*	76.9	23.1	
Regular ICS use	*Yes*	80.6	19.4	0.0401*
	*No*	72.4	27.6	
Follow up with clinics	*Yes*	77.8	22.2	0.5188
	*No*	75.1	24.9	
Education level	*High school or less*	77.2	22.8	0.3853
	*University*	72.1	27.9	
Educated about medication	*Yes*	78.6	21.4	0.2650
	*No*	74.0	26.0	
Educated about asthma	*Yes*	81.9	18.1	0.0117*
	*No*	71.9	28.1	

*The Chi-square statistic is significant at the .05 level.^*╦*^*All percentage rounded to one decimal.*

**Table 5:** The odds ratios and 95% CIs for the risk factors associated with three or more asthma-related ED visits

***Variable***	***levels***	**Estimate**	**P-value**	**OR**	**95% CI**	
Intercept		−0.2487	0.3982	-	-	-
Age		0.00344	0.5984	1.003	0.991	1.016
Gender	Female	0.0694	0.5192	1.149	0.753	1.752
Regular ICS use	No	0.0594	0.6348	1.126	0.690	1.838
ACT	Uncontrolled	0.3272	0.0063*	1.924	1.203	3.077
Follow up with clinics	No	−0.2746	0.0328*	0.577	0.349	0.956
Education level	University	0.4292	0.0071*	2.359	1.263	4.407
Educated about medication	No	0.0790	0.5844	1.171	0.665	2.062
Educated about asthma	No	0.2042	0.1506	1.504	0.862	2.625

* Wald Chi-square statistic is significant at the .05 level.

## Discussion

While this study is not epidemiological, it is the first study to investigate the factors leading to ED visits in a sample of Saudi bronchial asthma population and the characteristics of those patients. The major strength of this study lies in direct interviewing the patients and confirmation of the information obtained by reviewing the medical record. It is very important to examine these factors, because, we observed that many patients depend on the ED for asthma management. Knowing these factors may help address some of the deficiencies in our health system. The national and international guidelines for the management of bronchial asthma emphasise patient education and regular follow up with asthma professional. Our study generally showed that a substantial number of patients do not follow up asthma management with physicians and did not receive any education about asthma as a disease. A substantial number of our patients also used ED as an easy way to access their asthma management instead of keeping a follow up appointment with asthma professionals. This is not unique for our population, and many studies have reported the same findings [14,15,25]. The majority of our patients exhibited uncontrolled or partially controlled bronchial asthma (97.7%) in the months preceding the ED visit, which is unacceptably high. However, this result also consistent with our previous finding of a substantial percentage of uncontrolled or partially controlled bronchial asthma (95%) among the patients in major tertiary care hospitals [26]. The result of our study raises national concerns regarding our current asthma management system, which requires better health delivery structures, easy clinic access for patients, better patient education, better dissemination of the current national asthma guidelines and better monitoring. Asthma educators only educated 17% of the patients in this study; this was primarily due to the lack of trained asthma educators in many tertiary care hospitals and definitely contributes to the number of patients with uncontrolled asthma and the number of ED visits. The majority of our patients who had follow up visits (40%) attended the follow up at a primary care clinic, where the setting for asthma education is not very strong. The lack of patients education about asthma is obvious, as almost 40% of our patients were never taught how to use asthma devices. Studies have shown that ensuring that asthma patients understand their medication and the appropriate use of a drug delivery device contributes significantly to asthma control [27-30]. Furthermore, Hanania NA et al. [31] have shown that many of the medical personnel responsible for instructing and educating patients in optimal inhaler use lack rudimentary skills with these devices, seldom receive formal training in the use of inhalation devices, and may be not familiar with newer inhalation devices and techniques. We believe that our study identify probably a substantial problem in our health care system, particularly in the primary care setting. Abudahish, A et al. [32] have shown that asthma management in primary care is unsatisfactory. Our study also revealed the common misunderstanding of using the ED to receive a nebulised bronchodilator and oxygen as primary therapy for acute asthma among many of our patients. Approximately 80% of the patients were classified with mild asthma by the National Asthma Educating Program (NAEAP), and these patients would probably obtain relief from their symptoms by using rescue MDI bronchodilator without need to visit ED if they received the appropriate education. We also examined the factors that lead to three or more ED visits over the preceding year, believing that patients with frequent ED visits probably have less control over their asthma. In our study, the more educated patients reported three or more ED visits; however, the number of these patients was generally small (13%), and most of them experienced moderate to severe asthma (data not shown). Similar to other studies investigating the lack of asthma education, uncontrolled or partially controlled asthma were major reasons for the ED visit, in addition to inconsistent clinic visits [15,16]. This study is only based on two teaching hospitals in the central region of Saudi Arabia and may not reflect the situation at the national level. However, we believe that this study reflects the current general characteristics and risk factors for crisis oriented care and dependence on the ED for the management of bronchial asthma exacerbations. Furthermore, the situation may be even worse if we assessed these data at the country level, where the infrastructure for asthma management may be less well organised.

### Limitations

One of the major limitations of this study is the inability to assess the components or quality of the different asthma education or information programs our asthmatic patients received from health care professionals. In addition, we did not examine the detailed risk factors for asthma exacerbation, such as an environmental risk for exacerbations at home or in working environments. The second limitation is the lack of an economic evaluation for an ED visit. While the Saudi Arabian government provides free health care for all Saudi citizens, we could not readily assess the accessibility of outpatient clinics at both institutions, as it was not the aim of the study. Another limitation is not comparing the risk factors of our patients to those patients who attend outpatient clinics; however, our previous study found that the majority of the patients at outpatient clinics still have uncontrolled asthma [26] and hold many false beliefs and misconceptions about bronchial asthma as a disease and the role of inhaled corticosteroids and the factors affecting compliance among adult asthmatic patients [33].

## Conclusion

Our study has identified several factors that increase the risk of repeated ED visits for the crisis oriented care of asthma. The major factors we identified are a lack of asthma education, the lack of regular follow up with specialised asthma clinics, patient misunderstandings about the role of EDs in the treatment of bronchial asthma, and the underutilisation of inhaled steroid use. Most of these factors can be addressed by health care providers, and health care planners can rectify these problems by restructuring asthma management resources to emphasise a more multidisciplinary approach and invest in training additional asthma educators to participate in patient education and instruction of how to use inhaler devices and asthma action plans.

## Competing interests

The authors declare that they have no competing interests.

## Authors’ contributions

JH: Review the scientific literature pertinent to the research question. Writing the proposal and responding to reviewer and IRB comments. Create data collection form and draft the first manuscript. AA: perform all statistical analysis and writ the result section. HA: Supervising the data collection at KAMC. SB:-Scientifically contribute to writing the proposal. HR: Supervising the data collection at KKUH. SA : Providing scientific expertise and operational guidance to data collection at KAMC and actively precipitating in contributing in the manuscript writing as per assignment by PI. MS: Scientifically contribute to writing the proposal and study conduct at KKUH. All authors read and approved the final manuscript.

## Pre-publication history

The pre-publication history for this paper can be accessed here:

http://www.biomedcentral.com/1471-2466/12/80/prepub
